# Cancer-Associated Fibroblasts in Perineural Invasion: Biological Roles and Therapeutic Strategies

**DOI:** 10.3390/cells15201857

**Published:** 2026-10-13

**Authors:** Xinxing Cui, Yun He, Liu Yang, Aiping Lu, Fangfei Li

**Affiliations:** 1 Shum Yiu Foon Shum Bik Chuen Memorial Centre for Cancer and Inflammation Research (CCIR), School of Chinese Medicine, Hong Kong Baptist University, Hong Kong SAR, China; 2 Institute of Precision Medicine and Innovative Drug Discovery (PMID), School of Chinese Medicine, Hong Kong Baptist University, Hong Kong SAR, China; 3 Institute of Systems Medicine and Health Sciences (SMHS), School of Chinese Medicine, Hong Kong Baptist University, Hong Kong SAR, China

**Keywords:** cancer, perineural invasion, cancer-associated fibroblasts, immune suppression, neural microenvironment

## Abstract

Perineural invasion (PNI), the process of tumor cell infiltration of nerves, is a pathological feature of aggressive tumor behavior, correlating with therapeutic resistance and poor clinical outcomes. Cancer-associated fibroblasts (CAFs) have recently emerged as key regulators of PNI. CAFs drive PNI progression through multifaceted mechanisms, including dynamic remodeling of CAF subtypes, neural microenvironment remodeling, and enhancement of tumor–nerve crosstalk via neurotrophic signaling. While various therapeutic approaches targeting CAF surface markers, secreted factors, and intracellular signaling pathways are under investigation, none have yet been translated into clinical practice for PNI management. This review synthesizes current understanding of CAF-mediated PNI, highlights critical knowledge gaps, and proposes future directions. Here, we conceptualize CAF-mediated PNI as a coordinated process linking CAF subtype dynamics, neural niche remodeling, and tumor–nerve crosstalk, rather than as a collection of isolated pro-invasive mechanisms.

## 1. Background

Perineural invasion (PNI) is a pathological phenomenon in which tumor cells infiltrate and reside within the perineural space. This invasion is not just a passive spread of tumor cells, but a dynamic process driven by a complex molecular dialogue between tumor and nerve [1]. PNI is implicated in the poor prognosis of a variety of malignancies, including pancreatic cancers, head and neck squamous cell carcinoma (HNSCC), cutaneous squamous cell carcinoma, and colorectal cancer (CRC), and is closely associated with an elevated risk of local and regional recurrence and distant metastasis [2,3,4,5]. PNI carries significant clinical implications, correlating with diminished overall survival (OS), disease-specific survival (DSS), and disease-free survival (DFS). Its presence is a key determinant that can inform cancer staging and guide subsequent treatment decisions [6]. In addition, PNI can cause neurological symptoms, including pain, paresthesia, numbness, and paralysis [7].

Multiple cell types and molecular pathways participate in PNI. Tumor cells, immune cells, and nerve-associated cells jointly regulate tumor invasion along nerves. Tumor cells can secrete nerve-associated factors to support neurite outgrowth, while nerve-derived signals in turn enhance tumor cell migration and invasion [1,8,9]. Immune cells are a large group of cells in the tumor microenvironment (TME), and they can influence the process of PNI by influencing tumor cells [10]. For instance, M2 macrophages, regulatory T cells (Tregs), and neutrophils secrete IL-10, transforming growth factor beta (TGF-β), and CXCL12, thereby creating an immunosuppressive microenvironment that facilitates tumor survival and invasion [11,12,13,14]. Schwann cells (SCs), key components of the neural microenvironment, actively promote PNI by guiding tumor cell migration, remodeling the perineural extracellular matrix (ECM), and releasing neurotrophic signals [15]. These cellular interactions jointly promote the progression of PNI.

Cancer-associated fibroblasts (CAFs) are pivotal constituents of the TME that support tumor progression through multiple mechanisms. They secrete growth factors, cytokines and chemokines, including TGF-β and vascular endothelial growth factor, which contribute to the survival, proliferation and migration of tumor cells [16,17]. Additionally, CAFs can also remodel the ECM by producing and secreting matrix metalloproteinases (MMPs) and other matrix remodeling factors, thereby promoting the invasion of tumor cells [18,19]. Notably, CAFs also engage in bidirectional crosstalk with nerves. In pancreatic cancer, sympathetic signaling promotes CAF activation and ECM remodeling, while activated fibroblasts are linked to nerve injury-related changes in sympathetic neurons [20]. Through these processes, CAFs emerge as key players in tumor progression, metastasis, and immune evasion. Dissecting the interplay between CAFs and PNI, along with evaluating CAF-mediated prognostic implications, may offer new possibilities for developing novel therapeutic strategies aimed at blocking tumor–nerve interactions and improving patient outcomes.

A comprehensive literature search was conducted in PubMed, Web of Science, and Google Scholar databases using combinations of the following keywords: “perineural invasion”, “cancer-associated fibroblasts”, “tumor-nerve crosstalk”, “CAF subtypes”, and “therapy”. The search covered publications with emphasis on high-quality original articles, clinical studies, and systematic reviews published in peer-reviewed English-language journals. Reference lists of included articles were manually screened to identify additional relevant studies.

## 2. PNI as a Crucial Clinical Challenge in Cancer

### 2.1. Epidemiology and Clinical Impact of PNI

PNI is a process of tumor cells invading nerves. However, it should be noted that the diagnostic criteria for PNI are not entirely uniform across different studies. Some studies define PNI as tumor cells directly abutting a nerve and involving at least 33% of the neural circumference, whereas others consider the presence of tumor cells within any layer of the nerve sheath (epineurium, perineurium, or endoneurium) as PNI-positive [21]. Furthermore, the detection rate of PNI is influenced by factors such as the number of sections examined, the sampling plane, and the pathologist’s experience. Despite these variabilities, PNI is widely present in many malignant cancers, and the incidence of PNI varies among different cancer types. In pancreatic cancer, the incidence of PNI is 80–100%, and it is an important factor for local recurrence and disease progression [2]. In HNSCC, the cumulative incidence of PNI is 30% [5]. The incidence of PNI was 16.7% and 22.1% in patients with stage II and stage III CRC, respectively [3]. PNI is an established marker of poor prognosis, linked to reduced OS, DFS, and DSS, as well as increased recurrence and treatment resistance.

In pancreatic cancer, clinical data revealed that PNI-positive patients had a 2.24-fold higher risk of death compared to PNI-negative ones with N0-stage disease [22]. The median DFS for PNI-negative patients was 20 months, while that for PNI-positive patients was only 15 months [22]. Notably, neoadjuvant therapy was found to significantly reduce PNI incidence, indicating that early intervention might help mitigate its effects [22]. In HNSCC, a meta-analysis of 27,559 patients demonstrated that PNI decreased OS by 1.91-fold and DFS by 1.82-fold, while significantly increasing the risk of local recurrence and regional recurrence [5]. According to the AJCC 8th tumor staging system, PNI-positive lesions can increase the patient’s tumor stage from T2 to T3 [23]. Likewise, in CRC, a study of 1412 patients demonstrated a 5-year OS rate of 84.1% in PNI-positive patients, compared to 96.5% in PNI-negative ones [24]. Another study involving 371 stage II CRC patients indicated that PNI-positive individuals were nearly 4 times more likely to die and showed limited benefit from adjuvant chemotherapy, suggesting that PNI may confer intrinsic resistance to systemic therapy [3,24]. Moreover, PNI is a contributing factor to the development of neuropathic pain, a condition which has been found to result in a decline in patients’ quality of life and is associated with a worse prognosis [25]. Collectively, these findings highlight PNI as a critical determinant of disease progression, treatment response, and survival outcomes in multiple cancer types.

### 2.2. Key Cellular Players and Their Roles in the Process of PNI

PNI is a complex biological process involving multiple cell types within the TME, including tumor cells, immune cells, SCs and CAFs. Early research focused predominantly on tumor cells and their ability to invade and migrate along nerve structures. These studies highlighted tumor-derived neurotrophic signaling, such as nerve growth factor (NGF), and key chemokine pathways like CX3CL1/CX3CR1, which facilitate directed tumor cell migration toward and along nerves [26]. However, it is now recognized that PNI is not merely a tumor cell-autonomous process. Immune cells contribute to PNI by establishing a pro-tumor microenvironment that promotes neural invasion. For instance, tumor-associated macrophages and Tregs secrete cytokines like IL-6 and TGF-β, which induce epithelial–mesenchymal transition (EMT) and suppress anti-tumor immunity. These immune components not only enhance tumor cell invasiveness but also support nerve remodeling, aiding the progression of PNI [1]. Furthermore, and most critically, SCs, the principal glial cells of the peripheral nervous system, have been redefined from passive structural elements to essential participants in the PNI process. The “path of least resistance” hypothesis, originally thought to explain PNI, was expanded to recognize the reciprocal interactions between tumor cells and SCs. SCs facilitate tumor invasion by guiding cancer cells towards nerve fibers, secreting factors that remodel the ECM and promoting tumor cell survival [27]. This suggests that PNI is a dynamic and bidirectional process rather than a passive event.

Beyond the direct interactions between tumor cells and SCs, CAFs have emerged as key contributors to PNI, influencing both the tumor and nerve microenvironment. In a clinical and genetic study of nerve infiltration in HNSCC, researchers observed that CAFs often localize near partial EMT cells within the TME. Tumors exhibiting elevated CAF and partial EMT scores displayed a substantially heightened likelihood of invasion and metastasis [28]. Analysis of clinical samples revealed that PDPN^+^ CAFs are enriched in patients with PNI-positive pancreatic cancer [29]. Mechanistically, CAFs employ diverse strategies to promote PNI. They can directly influence tumor cells; for instance, pancreatic cancer-associated CAFs deliver the lncRNA perineural invasion-associated transcript (PIAT) via exosomes, which binds to YBX1 in cancer cells, inducing m5C alterations that drive a pro-PNI gene program [30]. Concurrently, CAFs reshape the neural environment. In a compelling example of intercellular crosstalk, CAFs in oral squamous cell carcinoma (OSCC) have been shown to suppress SC proliferation and migration by releasing leukemia inhibitory factor (LIF). This LIF-mediated inhibition reduces production of neurotrophic factors and myelin, thereby exacerbating nerve damage and facilitating the progression of PNI [31]. Given their central role, CAFs present a promising therapeutic target for disrupting PNI. Notably, the epigenetic enzyme EHMT2 was shown to govern CAF activation via H3K9 methylation. Inhibiting EHMT2 not only shifted the epigenetic profile of CAFs toward a more differentiated phenotype but also reduced the secretion of pro-invasive factors, thereby diminishing tumor cell invasion [32]. Collectively, these findings highlight CAFs as important organizers of the perineural tumor microenvironment, where they contribute to structural remodeling, stromal support, and the regulation of tumor–nerve interactions. Importantly, CAFs, SCs, immune cells, and neural components do not act independently during PNI, but instead form an interconnected cellular network within the perineural niche. These cell populations contribute to PNI through distinct yet complementary functions, collectively shaping a microenvironment that supports neural invasion and tumor progression.

## 3. The Roles of CAFs in PNI

### 3.1. Dynamic Remodeling of CAF Subtypes During Tumor Progression and Perineural Invasion

**Figure 1:**
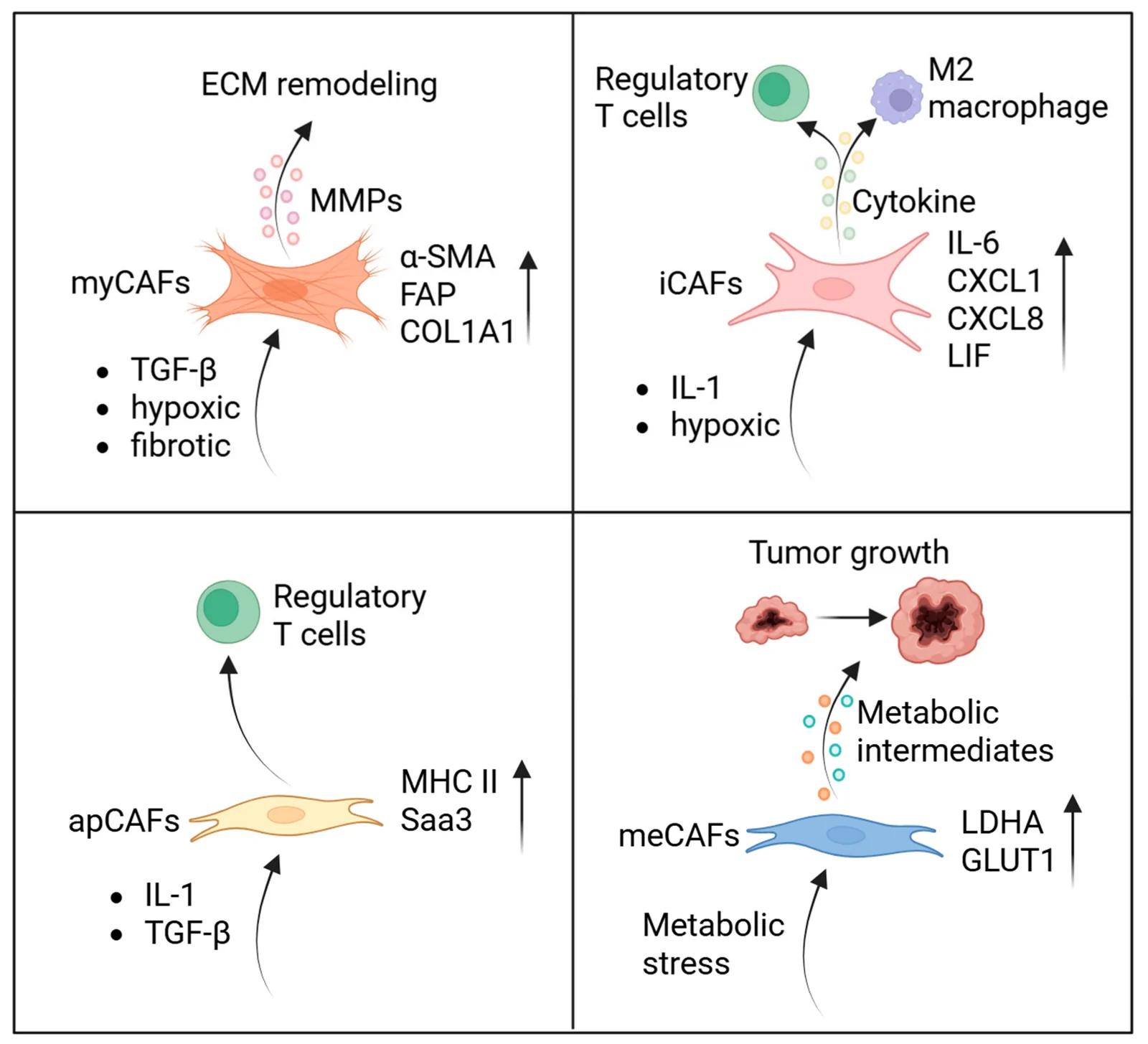
Dynamic remodeling and functional specialization of CAF subtypes during tumor progression and PNI. CAFs in the TME can be classified into several functional subtypes based on their stimuli and biological roles. myCAFs arise under TGF-β stimulation or in fibrotic and hypoxic conditions. They express high levels of α-SMA, FAP, and COL1A1 and remodel the ECM by secreting MMPs. iCAFs are induced by IL-1 and hypoxia, and they secrete pro-inflammatory cytokines including IL-6, CXCL1, CXCL8, and LIF, which contribute to immunosuppressive microenvironments by promoting M2 macrophage polarization and Treg induction. apCAFs express MHC II and Saa3, modulate T cell function, and may induce Tregs or impair T cell activation. They are regulated by IL-1 and TGF-β. MeCAFs respond to metabolic stress and facilitate tumor growth by producing metabolic intermediates and upregulating LDHA and GLUT1. Arrows indicate regulatory or functional relationships, upward arrows indicate increased expression, and colors distinguish CAF subtypes and associated cellular components. CAFs, cancer-associated fibroblasts; PNI, perineural invasion; myCAFs, myofibroblastic CAFs; iCAFs, Inflammatory CAFs; apCAFs, antigen-presenting CAFs; meCAFs, metabolic CAFs; ECM, extracellular matrix; MMPs, matrix metalloproteinases; α-SMA, alpha smooth muscle actin; FAP, fibroblast activation protein; COL1A1, collagen type I alpha 1 chain; IL, interleukin; CXCL, C-X-C motif chemokine ligand; LIF, leukemia inhibitory factor; MHC II, major histocompatibility complex class II; Saa3, serum amyloid A3; LDHA, lactate dehydrogenase A; GLUT1, glucose transporter 1.

CAFs are a heterogeneous group of stromal cells found within the TME of various cancers. These subsets dynamically respond to various signals in the TME, including those derived from tumors, immune cells, metabolism, and nerves. A recent study demonstrated that CAF composition differs according to nerve invasion status in human PDAC. Invaded nerve neighborhoods were enriched in myofibroblastic CAFs (myCAFs), whereas non-invaded nerve neighborhoods contained relatively more inflammatory CAFs (iCAFs) together with lymphocyte-rich populations. These PNI-specific spatial data support an association between CAF state and perineural invasion status, although the functional contribution of these distinct CAF populations remains to be established [33]. During tumor progression and PNI, different subsets may exert either pro-tumor effects, such as ECM remodeling, immune suppression, metabolic support, and neural niche formation, or anti-tumor effects, such as maintaining tissue homeostasis and promoting antitumor immunity (Figure 1). However, the extent to which these subtype-specific functions directly operate during PNI remains incompletely defined.

myCAFs are characterized by high expression of α-SMA (ACTA2), fibroblast activation protein (FAP) and ECM-associated genes such as COL1A1, markers associated with myofibroblast differentiation and ECM remodeling [34]. TGF-β signaling can promote myCAF differentiation, which is often associated with hypoxic and fibrotic conditions. Tumors with higher levels of fibrotic stroma, such as pancreatic cancer, breast cancer, and liver cancer, tend to have an elevated presence of myCAFs [35], although this general association does not necessarily indicate a PNI-specific role for myCAFs across these malignancies. Direct PNI-associated evidence has been reported in CRC, where single-cell and bulk transcriptomic analyses identified an MMP2^+^ myCAF population enriched in PNI-positive tumors, with PRRX2 acting as a key regulator that promotes perineural infiltration through TGF-β signaling and ECM remodeling [36]. In oral cavity squamous cell carcinoma, MMP-2-expressing fibroblasts were also detected in the perineural microenvironment, providing additional PNI-associated evidence for fibroblast-mediated matrix remodeling [37].

iCAFs, extensively characterized in pancreatic cancer, are a distinct CAF state characterized by relatively low expression of α-SMA and high secretion of inflammatory cytokines such as IL-6, CXCL1, CXCL8, and LIF [34]. Unlike myCAFs, which are closely associated with ECM remodeling, iCAFs primarily contribute to the shaping of an immunosuppressive and pro-tumor microenvironment, as demonstrated mainly in pancreatic and other non-PNI TME models. Through the release of cytokines, iCAFs can modulate immune cell infiltration and polarization, promote tumor cell survival, functions that may indirectly influence the perineural niche and potentially support PNI. In pancreatic cancer models, the induction of iCAFs is largely driven by IL-1 signaling, which activates the JAK/STAT pathway, and can be further influenced by hypoxic conditions within the TME [38]. Consistent with this inflammatory regulatory program, in PDAC, SCs can induce a shift toward an iCAF-like state through IL-1α, suggesting that iCAF-like states may participate in neuro-stromal remodeling [39]. In addition, LIF, an iCAF-associated cytokine, has been implicated in CAF-mediated SC dysfunction during PNI. In OSCC, CAF-derived LIF was reported to promote PNI by inducing SC inactivation, reducing SC proliferation and migration, disrupting myelin homeostasis, and aggravating nerve injury. However, because the LIF-expressing CAFs in this study were not explicitly annotated as canonical iCAFs, this finding should be interpreted as evidence for a LIF-dependent inflammatory CAF-like pro-PNI mechanism rather than definitive proof that iCAFs uniformly promote PNI [31].

Antigen-presenting CAFs (apCAFs) express MHC class II and serum amyloid A3 (Saa3), enabling them to interact with CD4^+^ T cells and influence immune responses [40]. Recent studies suggest that apCAFs originate from mesothelial cells, which, under IL-1 and TGF-β signaling during pancreatic cancer progression, lose mesothelial traits and acquire fibroblast-like characteristics [41]. Although not directly contributing to ECM remodeling, their immunoregulatory functions may theoretically influence the perineural niche. Their antigen-presenting capacity may induce T cell anergy or promote Treg differentiation, thereby suppressing immune surveillance in nerve-invasive tumors [35]. Whether this immunoregulatory function specifically facilitates immune evasion within the perineural niche remains to be determined.

Metabolically active CAF states, sometimes referred to as metabolic CAFs (meCAFs), are characterized by high expression of genes related to cellular metabolism, such as lactate dehydrogenase A (LDHA) and glucose transporter 1 (GLUT1) [42]. In PDAC, metabolically active CAF populations can contribute to tumor progression by producing metabolic intermediates, which serve as fuel sources for both cancer cells and immune cells, thereby supporting tumor growth and immune modulation [43]. Metabolic stress, such as glucose deprivation or hypoxia, can promote reprogramming of CAFs and the emergence of metabolically active CAF states. Additionally, tumors with high metabolic demands, such as those in liver, lung, and breast cancer, tend to have elevated meCAF populations [44], although these observations derive largely from general TME studies and do not establish a PNI-specific role for these CAF states across cancer types. Although direct PNI-specific evidence for canonical meCAFs remains limited, a recent PDAC study identified a high-lactate metabolic program in PNI-associated CAFs isolated from human PDAC samples. Importantly, these PNI-associated CAFs showed glycolytic upregulation and lactate accumulation, and CAF-derived lactate promoted PNI by inducing H3K18 lactylation in tumor cells and activating neural invasion-related genes, including L1CAM and SLIT1 [29].

In addition to the well-characterized myCAFs, iCAFs, and apCAFs, as well as metabolically reprogrammed CAF states, there are subtype-specific CAF populations in different cancer types. For instance, Lipid-Supplying CAFs have been identified in pancreatic cancer [42]. Similarly, studies in pancreatic cancer have identified several transcriptionally defined CAF populations, including ZEB1+ CAFs, GLI1+ HOXB6+ CAFs, SAA3+ CAFs, Complement-Secreting CAFs, and CD10+/GPR77+ CAFs [34]. In CRC, T-cell inhibitory CAFs promote immune evasion by upregulating checkpoint molecules and suppressing T-cell activity [45], although their direct contribution to PNI has not been established. Breast cancer models further illustrate fibroblast diversity, with distinct subgroups such as PDPN+ versus S100A4+ CAFs [46], but these breast cancer-defined populations have not been directly linked to PNI. Recent spatial transcriptomic studies further indicate that fibroblast heterogeneity within the perineural niche is spatially organized. In PDAC, a nerve-associated NRP2^+^ fibroblast population was identified within the endoneurial compartment and adjacent to SCs, indicating the presence of a fibroblast state spatially associated with the neural niche. Notably, non-invaded perineural regions were enriched in CXCL12^+^ fibroblasts, CXCR4^+^ B cells, and tertiary lymphoid structures, whereas invaded regions showed enrichment of myCAF programs and inflammatory macrophages [47]. These findings suggest that fibroblasts in the PNI microenvironment may have opposing functions, with some states associated with immune-rich, non-invaded neural niches and others associated with invaded, myofibroblast-enriched regions. Whether these spatially distinct fibroblast states directly exert opposing functional effects during PNI remains to be established.

Collectively, these examples underscore the dynamic remodeling of CAF subtypes and CAF-like states during tumor progression, which may influence processes such as immune suppression and PNI. Distinct CAF populations contribute to tumor progression and PNI by regulating ECM remodeling, inflammatory signaling, immune modulation, and metabolism regulation, whereas their specific contributions to PNI vary in strength of evidence across tumor types. Because the evidence linking individual CAF states to PNI varies across tumor types and experimental systems, Table 1 summarizes the principal CAF subtypes or CAF-like states discussed in this review, their representative markers, reported PNI-associated functions, PNI-specific evidence levels and key references. While the heterogeneity of CAFs has been extensively characterized in the context of general tumor biology, studies specifically examining CAF subtype dynamics during PNI remain scarce. Whether PNI-positive tumors harbor distinct CAF compositions, or whether existing CAF states acquire specialized neural niche-associated functions during tumor–nerve crosstalk, remains a critical unanswered question. Understanding how CAFs adapt within the PNI microenvironment is essential, as they may acquire unique phenotypic and functional traits that influence tumor–nerve interactions and neural invasion.

CAF populations not formally established as canonical CAF subtypes are described as CAF-like states or context-specific fibroblast populations. PNI evidence is categorized as direct PNI-specific evidence, PNI-associated evidence, or indirect/extrapolated evidence, according to whether findings are supported by functional PNI models, spatial/clinical association with the perineural niche, or mainly by broader TME/non-PNI studies.

### 3.2. CAF-Mediated Remodeling of the Neural Microenvironment in PNI

**Figure 2:**
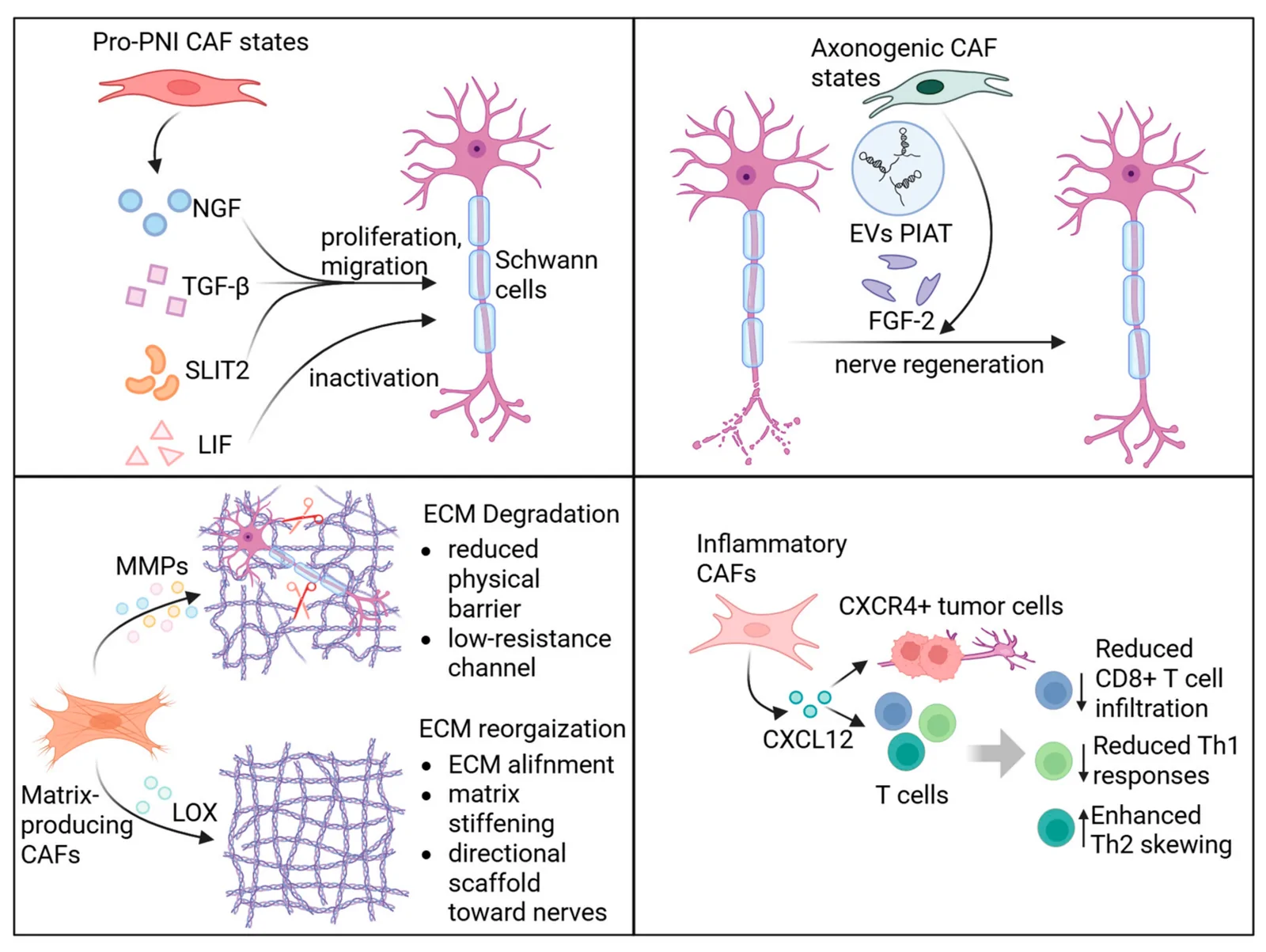
CAF-mediated remodeling of the neural microenvironment during perineural invasion. CAFs remodel the perineural niche through coordinated effects on SCs, axonal regeneration, ECM organization, and local immune exclusion. Pro-PNI CAF states secrete neurotrophic and stromal factors, including NGF, TGF-β, SLIT2 and LIF, which regulate SC proliferation, migration, and functional states. Axonogenic CAF states further promote nerve regeneration through factors such as FGF-2 and EV-associated PIAT. Matrix-producing CAFs remodel the perineural ECM through proteolytic degradation and mechanical reorganization: CAF-derived MMPs reduce physical barriers and generate low-resistance channels for tumor invasion, whereas LOX-mediated matrix stiffening and alignment may provide directional scaffolds toward nerves. Inflammatory CAFs also contribute to immune remodeling by producing CXCL12, which may promote CXCR4^+^ tumor-cell invasion while restricting effective T-cell infiltration. Together, these CAF-driven programs generate a permissive perineural microenvironment characterized by enhanced SC activity, nerve regeneration, matrix remodeling, and immune exclusion, thereby facilitating PNI. Arrows indicate the direction of signaling, regulatory, or functional relationships; the gray arrow denotes the resulting immune effects, while upward and downward arrows indicate increased and decreased responses, respectively. CAF, cancer-associated fibroblast; PNI, perineural invasion; SC, Schwann cell; ECM, extracellular matrix; NGF, nerve growth factor; TGF-β, transforming growth factor beta; SLIT2, slit guidance ligand 2; LIF, leukemia inhibitory factor; FGF-2, fibroblast growth factor 2; EV, extracellular vesicle; PIAT, perineural invasion-associated transcript; MMPs, matrix metalloproteinases; LOX, lysyl oxidase; CXCL12, C-X-C motif chemokine ligand 12; CXCR4, C-X-C motif chemokine receptor 4.

CAFs are key organizers of neural microenvironment remodeling during PNI. Distinct CAF states may collectively regulate SC proliferation and migration, axonogenesis and neo-innervation, perineural ECM remodeling, and immune compartmentalization within the perineural niche. These coordinated activities reshape the structural, neural, and immune landscape of the perineural microenvironment, thereby establishing a permissive niche for PNI (Figure 2).

#### 3.2.1. CAF-Induced Proliferation and Migration of SCs

CAFs can regulate SC activation, migration, and plasticity through the secretion of neurotrophic factors, axon guidance molecules, cytokines, and matrix-remodeling signals. These CAF-derived mediators may enhance SC survival, promote SC chemotaxis toward tumor cells, and facilitate the formation of SC-guided migratory tracks that support PNI. However, most current studies have examined CAF-SC crosstalk using bulk CAF populations or functionally defined PNI-associated CAFs, rather than resolving the contribution of individual CAF subtypes. Therefore, CAF-mediated regulation of SCs should be viewed as a heterogeneous and context-dependent process within the perineural niche.

CAFs secrete neurotrophic factors such as NGF [48], which can stimulate SCs via TrkA, enhancing SC motility and survival. NGF regulates the autophagy of SCs through the ATG7 pathway and enhances their chemotaxis to cancer cells, thereby promoting the infiltration of cancer cells along the nerves [49]. NGF also increases the chances of tumor cells invading around nerves by increasing the proliferation and migration of SCs [50]. SCs migrate into the tumor and form track-like structures between cancer cells, guiding tumor cells to infiltrate nerves. NGF-stimulated SCs secrete neurotrophic factors to promote the growth of nerve axons, which provides a pathway for tumor cells to invade and metastasize [15].

CAF-secreted axon guidance molecules may also regulate SC behavior. In PDAC, SLIT2 is expressed by CAFs and has been shown to enhance SC migration and proliferation through modulation of N-cadherin/β-catenin signaling. Inhibition of SLIT2/ROBO signaling disrupts this stromal–neural interaction, supporting a role for CAF-derived SLIT2 in SC regulation within the TME [51]. However, the effect of SLIT2 on SC migration appears to be context-dependent. In non-tumor models, SLIT2 has been shown to induce repulsive migration of SCs through Ca^2+^-dependent RhoA–myosin signaling [52]. Therefore, SLIT2 should be viewed as a context-dependent regulator of SC migration rather than as a uniformly pro-migratory factor. Consequently, while CAF-derived SLIT2 promotes SC migration and proliferation in PDAC, the question of whether SLIT2 exerts analogous or disparate effects on SCs in other tumor types remains unanswered and has not yet been systematically investigated.

Previous studies have shown that stromal fibroblasts, particularly CAFs, are a major source of transforming growth factor beta 1 (TGF-β1) in the TME [53]. CAF-derived TGF-β1 may therefore represent an important signal regulating SC behavior during perineural niche remodeling. Supporting this possibility, TGF-β1 has been shown to enhance SC migration and invasion by upregulating MMP-2 and MMP-9. Mechanistically, MMP-2 induction is mainly mediated by TGF-β receptor-SMAD2 signaling, whereas MMP-9 induction involves ERK1/2-JNK1/2-NF-κB activation [54]. Consistent with this, spatial omics profiling of pancreatic cancer with high neural invasion also revealed that myCAFs collectively surround invaded nerves and induce TGFBI^+^ SCs via TGF-β signaling, consequently promoting tumor cell migration and neural invasion [47].

LIF is a CAF-derived cytokine implicated in CAF-SC crosstalk during PNI. In OSCC, CAF-derived LIF promotes PNI by inducing SC inactivation and disrupting myelin homeostasis through increased endoplasmic reticulum stress and reduced MAPK signaling. Pharmacological inhibition of LIF reversed CAF-induced SC inactivation, indicating that LIF is a key mediator by which CAFs impair SC function. Thus, unlike NGF or TGF-β1, CAF-derived LIF may promote PNI mainly by aggravating nerve injury and disrupting SC homeostasis rather than by enhancing SC migration [31]. Beyond soluble-factor-mediated crosstalk, recent evidence indicates that the fibrotic mechanical microenvironment can also regulate SC activation in PDAC. Nerves embedded within stiffer stromal regions showed increased SC activation, while mechanical forces directly induced c-Jun phosphorylation through a nuclear mechanosensing mechanism. These findings suggest that stromal fibrosis may provide an additional mechanical route through which the CAF-rich microenvironment influences SC behavior during PNI [55].

While CAFs have been shown to promote SC activation, recent advances in cellular reprogramming raise an intriguing but still speculative question: could CAFs themselves acquire neuroglial-like features within the TME [56]? Evidence from regenerative and non-cancer experimental systems indicates that fibroblasts can be induced to adopt Schwann-like phenotypes in vitro through transcription factors such as SOX10 and KROX20 or through small-molecule cocktails [57,58,59]. Although these findings are limited to non-cancer contexts, they suggest a latent plasticity in fibroblasts that may, under certain tumor conditions, contribute more directly to nerve remodeling or perineural invasion. Whether such reprogramming occurs in CAFs in vivo remains unknown and merits further investigation.

Collectively, CAF-derived signals can regulate SC proliferation, migration, chemotaxis, and plasticity, thereby facilitating PNI. Nevertheless, the CAF subtype-specific origins of NGF, SLIT2, TGF-β, LIF, and other SC-regulatory mediators remain incompletely defined. Future studies combining spatial transcriptomics, ligand–receptor analysis, and functional validation are needed to determine which CAF states are responsible for SC activation within the perineural niche.

#### 3.2.2. CAF-Driven Axonogenesis, Neo-Innervation, and Neuronal Remodeling

Nerve regeneration is generally considered a physiological repair response after neural injury. However, in tumors, similar regenerative programs can be hijacked to promote axonogenesis, neo-innervation, and PNI. This hijacking process involves various nerve subtypes, including sympathetic, parasympathetic, and sensory nerves, which are known to play distinct and sometimes opposing roles in tumor progression.

CAFs play a key role in nerve repair and regeneration through the secretion of fibroblast growth factor (FGF), especially basic FGF (bFGF/FGF2) [60]. By activating the FGF/FGF receptor signaling pathway, these growth factors promote neuronal survival, sensory axonal growth, SC proliferation and myelin sheath formation, which are essential steps in the recovery of damaged nerves [61,62]. In dorsal root ganglia (DRGs), bFGF expression increased after axotomy, suggesting a potential role in neuronal survival and regeneration [63]. Similarly, in sciatic nerve injury models, exogenous FGF2 application effectively promoted myelin regeneration, increased nerve conduction velocity, and accelerated functional recovery [64]. Although these studies were largely conducted in nerve injury rather than tumor-specific models, they suggest that fibroblast-associated regenerative factors may be co-opted in the TME to generate permissive neural structures for PNI.

In addition, CAF-derived extracellular vesicles (EVs) have been reported to transfer the long non-coding RNA PIAT and promote PNI-associated neural remodeling in pancreatic cancer. In vitro DRG co-culture assays and mouse sciatic nerve models demonstrated that CAF-derived EVs contribute to PNI. Furthermore, KPC mouse models supported the role of the PIAT/YBX1 axis in promoting PNI through m^5C-dependent mechanisms. Mechanistically, EV-packaged PIAT binds YBX1, blocks Nedd4l-mediated YBX1 ubiquitination and degradation, and stabilizes YBX1. Stabilized YBX1 enhances the recognition of m5C-modified PNI-associated transcripts, thereby promoting neural remodeling in models of pancreatic cancer. From a clinical perspective, elevated PIAT levels in serum EVs from individuals diagnosed with pancreatic cancer were associated with more extensive neural invasion and a poorer prognosis. However, the patient data are associative rather than causal, and most mechanistic evidence was obtained from co-culture systems and mouse models. Consequently, the PIAT/YBX1 axis can be interpreted as a promising, albeit preclinical, mechanism of CAF-tumor–nerve communication. The broader relevance of this axis across tumor types remains to be established [30]. Taken together, the available evidence indicates that CAFs can interact with sensory neurons to promote axon regeneration, neurite outgrowth, and nerve fiber remodeling, thereby potentially facilitating PNI.

#### 3.2.3. CAF-Mediated Remodeling of the Perineural ECM

CAFs promote PNI by remodeling the perineural ECM to facilitate tumor cell-nerve interactions. In high-PNI distal cholangiocarcinoma tissues, matrix-producing CAFs are enriched and exhibit elevated expression of fibrosis- and ECM remodeling-associated genes (such as MMP14, SULF1, CTHRC1, and THBS2), consistent with a matrix-remodeling stromal environment associated with neural invasion [65]. Tumor cells need to penetrate the ECM around the nerves to achieve perineural infiltration, and MMPs provide tumor cells with a channel for migration and invasion by degrading ECM [66]. MMP-2 and MMP-9 secreted by CAFs can degrade the ECM around the nerves, including the basement membrane of the epineurium and endoneurium, reducing the physical barriers for tumor cells to invade the nerves, thereby forming a low-resistance channel, helping tumor cells to infiltrate along the nerve fibers and eventually invade the nerve bundles, leading to PNI [19,37]. Recent single-cell and functional analyses identified MMP2^+^ myCAFs as a PNI-associated CAF subset enriched in PNI-positive tumors. PRRX2 acts as a key regulator of this population, promoting ECM remodeling and perineural infiltration through TGF-β signaling while also modulating TGF-β1 and NGF expression [36]. In addition, stromal fibroblasts can release MMP-2 in its inactive pro-form, which can be activated by tumor-cell-associated MT1-MMP, thereby promoting basement membrane remodeling and tumor invasion [67].

However, CAF-mediated ECM remodeling in PNI is not limited to matrix degradation. Based largely on evidence from the broader TME, myCAFs can physically pull and reorganize matrix fibers, converting a disordered ECM into aligned collagen and fibronectin bundles [68]. In addition, CAF-derived crosslinking enzymes such as lysyl oxidase (LOX) further stabilize collagen fibers and increase matrix stiffness [69]. The resulting stiffened and aligned ECM may act as a directional scaffold that facilitates tumor cell migration toward nerves and increases tumor–nerve contact. Tumor cells can then sense these CAF-remodeled mechanical cues through mechanosensitive pathways, including integrin-FAK, Piezo1, Rho-ROCK, PI3K-AKT, and Hippo-YAP/TAZ signaling, thereby enhancing motility and invasive behavior [70,71,72]. Therefore, CAFs may promote PNI not only by degrading ECM barriers, but also by constructing a mechanically permissive perineural niche that supports neural invasion.

#### 3.2.4. CAF-Driven Immune Exclusion in the Perineural Niche

The perineural compartment is increasingly recognized as a neuro-immune niche rather than merely an anatomical route for tumor dissemination, as PNI in PDAC is associated with impaired antitumor immunity, including reduced CD8^+^ T-cell and Th1 responses and enhanced Th2 skewing [73]. Direct evidence that CAFs specifically organize immune exclusion within this nerve-associated compartment remains limited; however, spatial and single-cell analyses show that SC-rich regions in PDAC are associated with iCAF signatures [39]. Mechanistically, SCs can promote the transition of CAFs toward an iCAF-like state through IL-1α, suggesting that nerve-associated glial cells may reprogram nearby CAFs into inflammatory stromal populations [39]. These findings support an emerging model in which nerve-associated CAF states may participate in local immune exclusion by integrating neuro-stromal crosstalk with established CAF-mediated immunomodulatory programs in the broader TME.

CAF-driven immune exclusion in the perineural niche may be mediated by the CXCL12-CXCR4 axis, one of the key stromal signals that also links CAF activity to neural invasion [74]. CAF-derived circKLHL24 enhances CXCL12 signaling by stabilizing Sec31a-mediated vesicular trafficking and relieving miR-615-5p-mediated transcriptional repression, thereby establishing a neurotrophic niche that promotes CXCR4-dependent tumor invasion and nerve outgrowth [75]. This pro-PNI function is conceptually linked to the established role of FAP^+^ CAF-derived CXCL12 in spatial immune exclusion, where stromal CXCL12 restricts T-cell access to cancer cells and CXCR4 blockade enhances T-cell infiltration and sensitizes tumors to checkpoint blockade [76]. Thus, CAF-derived CXCL12 may help generate a pro-tumor perineural microenvironment that supports both tumor–nerve crosstalk and immune evasion, although direct evidence integrating these two functions within nerve-associated regions remains limited.

Other iCAFs-associated mediators, including IL-6, LIF and CXCL1, may further contribute to inflammatory and myeloid-biased immune remodeling, but their roles in perineural immune exclusion should be interpreted mainly from broader TME studies rather than PNI-specific evidence [77]. Thus, CAF-driven immune exclusion in the perineural niche should currently be viewed as an emerging model in which nerve-associated CAF states, CXCL12-dependent spatial exclusion, iCAF inflammatory cytokines and neuroimmune suppressive signaling may cooperate to generate a locally tumor-permissive immune microenvironment.

More broadly, SCs contribute primarily to migratory guidance and neural remodeling. Immune cells shape inflammatory and immunosuppressive conditions, while neuronal components provide neurotrophic and neurotransmitter-derived cues. CAFs, on the other hand, integrate stromal, mechanical, metabolic and paracrine signals. Rather than functioning in isolation, these populations engage in reciprocal and cooperative interactions that collectively promote PNI progression.

### 3.3. CAF-Induced Tumor–Nerve Interactions

**Figure 3:**
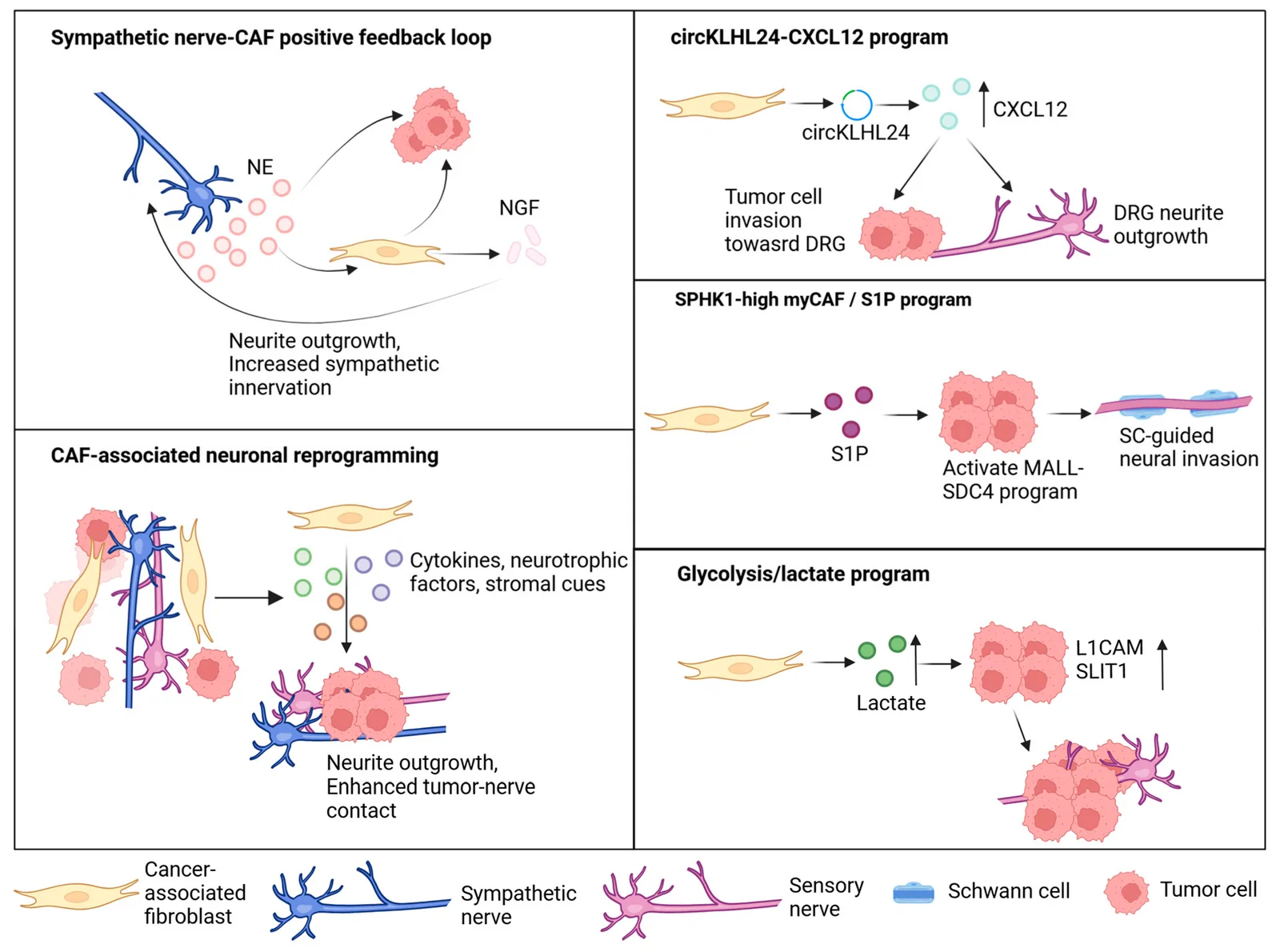
CAF-induced tumor–nerve interactions. CAFs promote PNI through multiple signaling programs that enhance tumor–nerve crosstalk. Sympathetic nerve-derived NE induces CAF-derived NGF, forming a positive feedback loop that enhances neurite outgrowth and sympathetic innervation. CAF-associated stromal cues may also contribute to neuronal reprogramming, leading to increased neurite outgrowth and tumor–nerve contact. CAF-derived circKLHL24 promotes CXCL12 signaling to support tumor cell invasion toward DRG and DRG neurite outgrowth. A SPHK1-high myCAF subset secretes S1P, which activates a MALL-SDC4 program in tumor cells and promotes SC-guided neural invasion. In addition, CAF glycolytic reprogramming generates lactate, which induces neural invasion-associated genes such as L1CAM and SLIT1 in tumor cells. Together, these mechanisms amplify tumor–nerve crosstalk and facilitate PNI. Arrows indicate the direction of signaling, regulatory, or functional relationships, whereas upward arrows indicate increased expression or levels of the indicated molecules. CAF, cancer-associated fibroblast; PNI, perineural invasion; NE, norepinephrine; NGF, nerve growth factor; KLHL24, kelch-like family member 24; CXCL12, C-X-C motif chemokine ligand 12; DRG, dorsal root ganglion; SPHK1, sphingosine kinase 1; S1P, sphingosine-1-phosphate; MALL, mal-like protein; SDC4, syndecan 4; L1CAM, L1 cell adhesion molecule; SLIT1, slit guidance ligand 1.

CAFs not only remodel the neural microenvironment but also serve as active intermediaries that strengthen functional communication between tumor cells and nerves. CAF-driven neurotrophic and neurotransmitter-associated feedback loops, circRNA-chemokine signaling, lipid signaling, and metabolic remodeling can promote directed tumor invasion toward neural structures, SC-associated neural invasion, and reinforced tumor–nerve communication during PNI (Figure 3).

CAFs can strengthen tumor–nerve interactions by participating in neurotrophic and neurotransmitter-associated feedback loops. In CRC, sympathetic nerves release NE, which acts on tumor cells and activates β2-adrenergic receptor signaling in CAFs, thereby inducing CAF-derived NGF production. This NE-CAF-NGF circuit increases intratumoral sympathetic innervation and norepinephrine (NE) accumulation, ultimately promoting tumor development and neural regulation [48]. CAF-derived NGF promotes neurite outgrowth by activating Trk receptors on nerve cells and may further reinforce neurotransmitter-associated tumor–nerve communication [78,79]. The functional relevance of NGF-Trk signaling is supported by studies showing that Trk inhibition suppresses neurite growth and attenuates neurotrophic signaling [80]. Thus, this CAF-sympathetic nerve feedback circuit may contribute to tumor progression in CRC by increasing sympathetic innervation. By contrast, the interaction between parasympathetic nerves and CAFs remains largely unexplored. Direct evidence for parasympathetic-CAF crosstalk in PNI is currently limited, and the underlying mechanisms and functional implications warrant extensive future investigation.

Beyond individual neurotrophic or axon-guidance factors, CAFs may also contribute to the functional remodeling of tumor-innervating peripheral neurons. A recent Trace-n-Seq study characterized PDAC-innervating sympathetic and sensory neurons at single-cell resolution and identified a pancreatic cancer nerve signature involving neuronal development, axon guidance, metabolic remodeling and microenvironmental interaction programs. Integrative ligand–receptor analysis further showed that CAFs are among the strongest predicted interaction partners of PDAC-innervating CG and DRG neurons, suggesting that CAF-derived or CAF-associated signals may participate in shaping the neural compartment [81]. In addition, together with functional evidence that sensory neurons promote PDAC initiation and progression and that sensory nerve endings can form glutamatergic pseudo-synapses with PDAC cells, these findings support a model in which CAF-neuron crosstalk may help generate a permissive neural compartment for tumor–nerve interaction and PNI [82,83].

Recent studies in pancreatic cancer have further expanded CAF-mediated tumor–nerve interactions beyond classical cytokine and neurotrophic signaling. One study showed that CAF-derived circKLHL24 enhances CXCL12 secretion through the Sec31a axis, thereby promoting tumor cell invasion toward the DRG and DRG neurite outgrowth [75]. Another study identified a SPHK1-high myCAF-like subset that secretes sphingosine-1-phosphate, which activates a MALL-SDC4 program in cancer cells to enhance amoeboid motility and SC-guided neural invasion [84]. Additionally, CAFs upregulate glycolysis to generate a high lactate microenvironment, which induces histone H3K18 lactylation in tumor cells and activates neural invasion genes such as L1CAM and SLIT1, thereby driving PNI [29]. These findings suggest that CAF-driven non-coding RNA and metabolic programs represent distinct routes by which CAFs amplify tumor–nerve crosstalk.

**Figure 4:**
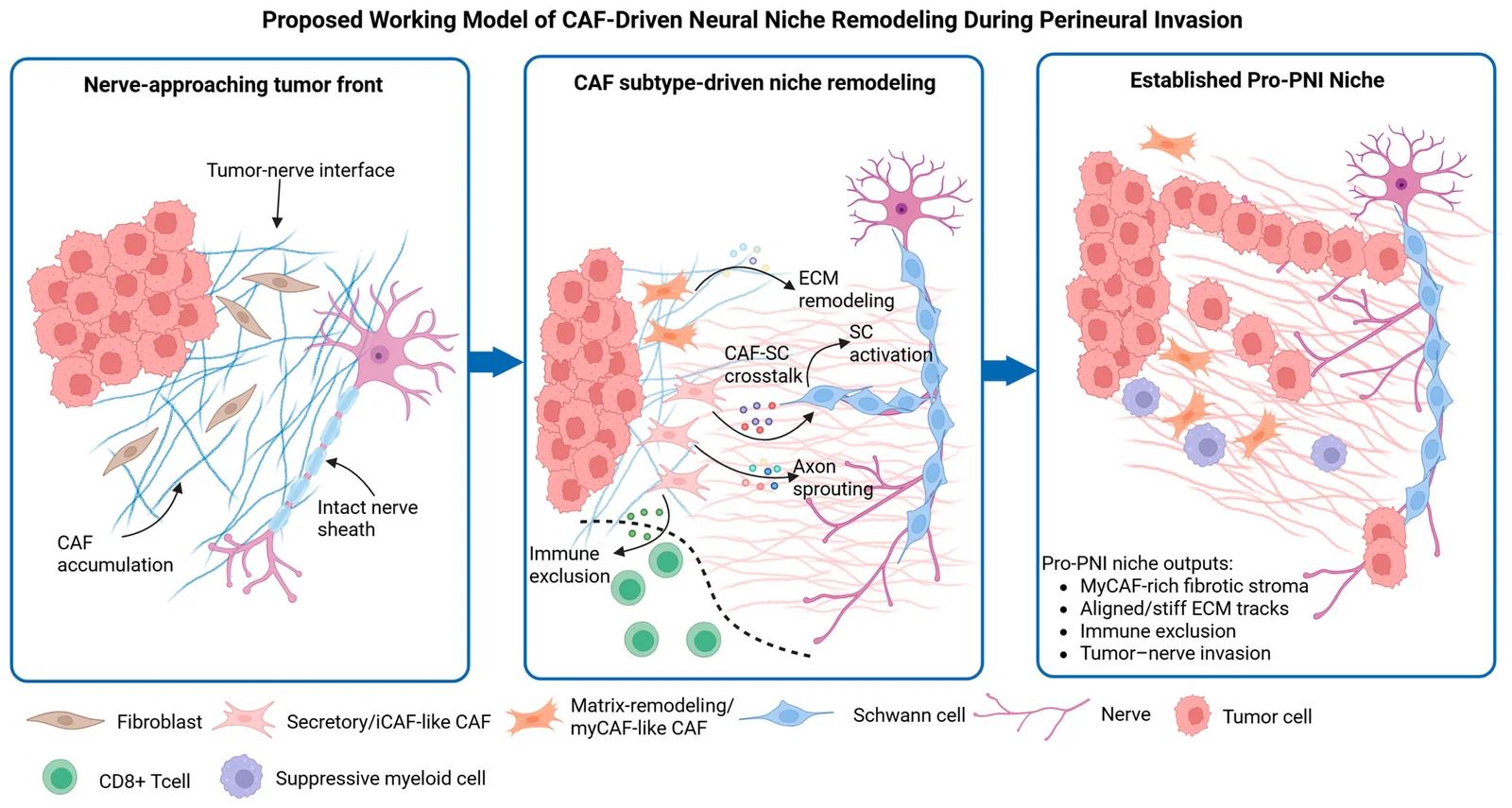
Proposed working model of CAF-driven neural niche remodeling during PNI. The schematic illustrates a proposed progression by which CAFs remodel the tumor–nerve interface into a PNI niche. At the nerve-approaching tumor front, CAFs begin to accumulate between tumor cells and an intact nerve sheath. During CAF subtype-driven niche remodeling, secretory/iCAF-like CAFs and myofibroblastic/myCAF-like CAFs contribute to CAF-SC crosstalk, SC activation, axon sprouting, ECM remodeling, and immune exclusion. These coordinated stromal, neural, and immune changes promote formation of an established pro-PNI niche characterized by myCAF-rich fibrotic stroma, aligned/stiff ECM tracks, reduced CD8^+^ T-cell access, suppressive myeloid cell accumulation, and tumor–nerve invasion. Black arrows indicate regulatory or functional relationships, blue arrows indicate the proposed progression between stages, and the dashed line denotes the boundary of the immune-excluded region. CAF, cancer-associated fibroblast; PNI, perineural invasion; iCAF, inflammatory cancer-associated fibroblast; myCAF, myofibroblastic cancer-associated fibroblast; SC, Schwann cell; ECM, extracellular matrix; CD8^+^ T cell, CD8-positive T cell.

Based on the evidence reviewed above, we propose a possible working model in which CAF subtype dynamics spatially and temporally remodel the tumor–nerve interface into a pro-PNI neural niche. Secretory/iCAF-like and matrix-remodeling/myCAF-like programs may cooperate to integrate SC activation, axonal remodeling, ECM remodeling, immune exclusion, and tumor–nerve crosstalk, thereby facilitating PNI (Figure 4).

### 3.4. CAF-Induced PNI-Associated Cancer Pain

The role of CAFs in promoting PNI has been well-established, and the clinical impact of PNI often manifests as pain, paresthesia, and paralysis [85]. Recent studies have revealed a positive feedback loop that amplifies pain sensation, involving sensory neurons and CAFs. In PDAC, CAF-derived SLIT2 has been shown to stimulate neurite outgrowth from DRG neurons [51]. Furthermore, nociceptive neurons interact with CAFs to accelerate tumor growth and exacerbate cancer pain. Mechanistically, the release of calcitonin gene-related peptide (CGRP) from nociceptor neurons is promoted by the secretion of NGF from CAFs [86]. Elevated CGRP directly contributes to the generation of pain [87]. Furthermore, NGF upregulates multiple pain-related genes in DRG sensory neurons (such as substance P, TRPV1, and Nav1.8), suggesting that CAF-derived NGF may modulate neuropathic pain through multiple mechanisms [88,89,90]. It is important to note that this crosstalk between fibroblasts and sensory neurons is not exclusive to cancer. In the context of rheumatoid arthritis, the conditioned medium of synovial fibroblasts has been observed to promote neurite branching of CGRP+ DRG neurons in vitro [91]. As previously stated, a distinguishing characteristic of iCAFs is their elevated level of IL-6 secretion. Research has demonstrated that the administration of an IL-6–neutralizing antibody can result in a reduction in neuropathic pain [92]. Furthermore, the secretion of PI16 by fibroblasts surrounding the peripheral nervous system and central nervous system has been identified as a regulator of neuropathic and inflammatory pain [93,94]. Collectively, these findings demonstrate that CAFs may actively contribute to PNI-associated cancer pain through multiple mechanisms. The targeting of these CAF-driven pathways has the potential to provide novel strategies for the management of cancer pain, while concurrently impeding PNI.

## 4. Current Therapeutic Strategies for Targeting CAFs

Targeting CAFs has emerged as a promising strategy to disrupt the tumor-supportive microenvironment. Current therapeutic approaches focus on depleting CAFs, inhibiting their activation, or blocking CAF-derived signaling pathways involved in tumor progression, immune suppression, and PNI. Although several strategies have shown efficacy in preclinical models, clinical translation remains challenging due to CAF heterogeneity and their functional plasticity. Despite substantial preclinical evidence, CAF-targeted approaches specifically for PNI have not yet been established as standard clinical therapies (Table 2).

Evidence labels distinguish direct testing in a PNI model, PNI-related mechanistic or cellular evidence, and indirect evidence extrapolated from broader CAF or cancer studies. Pathway involvement in PNI does not establish anti-PNI efficacy for every agent targeting that pathway. Development stages refer to the listed indications, not to PNI treatment.

### 4.1. Targeting CAF Surface Markers

Among surface markers, FAP is the most widely studied CAF target. FAP-expressing CAFs promote ECM fibrosis and secrete neural invasion-related factors such as NGF and MMP-2, thereby facilitating PNI. Pharmacological inhibition of FAP can suppress CAF activity. For instance, the FAP inhibitor Val-boro-Pro (Talabostat) showed ECM-degrading and tumor growth-suppressive effects in preclinical models, though a Phase II clinical trial in CRC demonstrated limited benefit, highlighting the need for improved strategies [95]. Another therapeutic approach involves FAP-targeted immunotoxins, which have been shown to suppress breast tumor growth and enhance immune infiltration in preclinical models [96]. In addition, FAP-targeted tracers combined with PET/CT imaging enable more accurate tumor staging and can detect metastatic lesions [115].

Platelet-derived growth factor receptor-beta (PDGFR-β) is prominently expressed on CAFs, playing key roles in tumor-stroma crosstalk and tumor progression [116]. Multiple tyrosine kinase inhibitors can block PDGFR signaling, but their lack of specificity limits translational impact [97]. Notably, the PDGFRα-targeting monoclonal antibody olaratumab initially received accelerated FDA approval for soft tissue sarcoma [98], but this indication was later withdrawn after the ANNOUNCE phase III trial failed to confirm clinical benefit [99]. This highlights the translational uncertainty of targeting stromal growth factor pathways.

Beyond small-molecule inhibition, immunotoxins, and imaging-based approaches, FAP-targeted immunotherapy has emerged as an additional strategy to modulate CAF-rich tumor stroma. Preclinical studies of FAP-targeted CAR-T cells suggest that targeting FAP^+^ stromal cells can disrupt tumor-supportive stroma and enhance antitumor immunity [117]. Subsequent studies have further evaluated FAP scFv-based CAR-T cells, bispecific CAR-T cells targeting FAP and tumor antigens, and mRNA-LNP-based in vivo CAR delivery [118,119,120]. However, these strategies have not been tested specifically in PNI models, and potential on-target/off-tumor toxicity remains an important concern. Therefore, FAP-directed immunotherapy should be viewed as a promising but indirect approach for remodeling CAF-rich stromal niches that may support PNI.

### 4.2. Targeting CAF-Derived Secreted Factors

CAFs secrete a variety of signaling molecules that create a permissive microenvironment for tumor cells to invade nerves. These mediators can be broadly categorized into neurotrophic chemokines, immune/EMT regulators, EVs, and matrix remodeling factors.

NGF and CCL2 are key mediators promoting tumor–nerve interactions via receptor-mediated signaling pathways (such as NGF/TrkA, CCL2/CCR2). Experimental inhibition of the YAP1/TEAD1–NGF/CCL2 circuit supports its involvement in PNI [109]. In pancreatic cancer, the CCR2 inhibitor PF-04136309 combined with FOLFIRINOX showed encouraging tumor control in a non-randomized phase Ib study, without PNI-specific efficacy assessment [100]. However, clinical attempts to target CCL2, such as with the monoclonal antibody Carlumab, showed limited efficacy in prostate cancer, underscoring the difficulty of fully suppressing this axis in patients [101].

IL-6 and TGF-β secreted by CAFs contribute to immune suppression and EMT induction, reinforcing invasive tumor phenotypes. Tocilizumab, an IL-6 receptor antibody, has shown antitumor activity in CRC xenografts [102]. TGF-β inhibitors, such as Galunisertib, can enhance the activity of immunotherapies in preclinical settings [103], and SB-431542 has been shown to inhibit SC-induced migration and invasion of pancreatic cancer cells by blocking TGF-β signaling [104]. Although TGF-β inhibitors have shown potential in preclinical studies, they have not achieved significant success in clinical trials. This may be related to the complex role of TGF-β in the TME and the adaptability of tumor cells.

Recent work highlights CAF-derived EVs as critical mediators of neural remodeling. In pancreatic cancer, CAF-secreted EVs deliver PIAT, which stabilizes YBX1 and facilitates the expression of PNI-associated genes through m5C modification [30]. Targeting EV biogenesis or disrupting the PIAT-YBX1 axis may therefore provide novel strategies to limit PNI.

During PNI-positive OSCC, CAFs impair SC proliferation and neurotrophic activity through the LIF-LIF receptor (LIFR) pathway, thereby compromising nerve repair and favoring PNI progression. Pharmacological blockade of this axis, for example with the LIF inhibitor EC330, can restore SC proliferation and migration in cellular and organotypic assays; direct drug-mediated reduction in PNI in vivo remains to be established [31,105]. The therapeutic relevance of LIFR inhibitors in this context warrants further study.

LOXL2 enhances ECM crosslinking and CAF motility, contributing to a pro-invasive microenvironment. Although its direct role in PNI has not been validated, ECM stiffening and alignment likely facilitate tumor cell migration along nerves [121]. Simtuzumab, a LOXL2-targeting monoclonal antibody, showed tolerable safety and occasional tumor shrinkage in Phase I/Iia trials among patients with advanced solid tumors [106]. However, two randomized Phase II trials, one testing Simtuzumab with FOLFIRI in metastatic KRAS-mutant CRC and the other testing Simtuzumab with Gemcitabine in pancreatic adenocarcinoma, failed to show clinical benefit in key efficacy endpoints such as PFS or OS [107,108].

In summary, targeting CAF-secreted factors offers multiple therapeutic opportunities, but clinical translation is hampered by pathway redundancy and tumor adaptability. Combining inhibitors of CAF-derived signals with immunotherapies or chemotherapy may provide a more effective strategy to suppress PNI.

### 4.3. Targeting CAF-Activated Intracellular Signaling Pathways

Several intracellular signaling pathways in CAFs have emerged as promising therapeutic targets in the context of tumor progression and potentially PNI. YAP/TEAD signaling has been implicated in prostate cancer, where CAFs promote PNI through a YAP1/TEAD1–NGF/CCL2 feedback loop that drives EMT and tumor–nerve invasion [109]. Separately, YAP/TEAD inhibition with Verteporfin has been shown to reduce tumor adhesion, migration, and ECM remodeling in glioblastoma models, highlighting the therapeutic potential of YAP/TEAD inhibition [110].

JAK2/STAT3 is a central axis in CAFs that sustains their activated state and promotes tumor invasion. In gastric cancer, CAF-derived IL-17a activates JAK2/STAT3 within tumor cells, enhancing invasion and correlating with poor prognosis and PNI [111]. Preclinical STAT3 intervention studies in non-small cell lung cancer and breast cancer support effects on CAF–tumor crosstalk, but do not directly establish inhibition of neural invasion [112,113]. Although several STAT3/JAK2 inhibitors, such as Napabucasin, TTI-101 and Fedratinib, have been tested in clinical trials for solid tumors, their role in CAF-driven PNI remains unexplored.

PI3K/AKT pathway, central to CAF metabolic activity and tumor–stroma communication, is also under investigation. Alpelisib, an FDA-approved PI3Kα inhibitor for HR+/HER2– breast cancer, has not yet been studied in the context of PNI, despite its relevance to CAF function. The allosteric AKT inhibitor MK-2206, evaluated in early-phase trials for various solid tumors, has been shown to promote invasion and metastasis in selected KRAS- or EGFR-mutant non-small cell lung cancer models, though without direct links to PNI [114].

The major CAF-targeted therapeutic strategies discussed above, together with their targets, clinical indications, and levels of PNI-related evidence, are summarized in Table 2.

### 4.4. Safety Considerations and Lessons from Stromal Depletion

Despite the promise of CAF-targeted treatments, safety concerns associated with aggressive stromal depletion should be carefully considered. A key lesson comes from FAP-directed strategies. Although these treatments effectively eliminate FAP^+^ CAFs in preclinical models, their clinical use has generated concerns about on-target/off-tumor toxicity, as FAP is also expressed at low levels on normal cells, such as bone marrow stromal cells and mesenchymal progenitors [118]. Preclinical research has shown that administering FAP CAR-T might cause fatal osteotoxicity and cachexia, underscoring the possibility of serious, unintentional tissue damage when FAP-expressing normal cells are also targeted. Similarly, the FAP-targeted IL2 variant FAP-IL2v (simlukafusp alfa) has been linked to treatment-related side effects in patients with advanced solid tumors, such as infusion-related reactions, hypotension, capillary leak syndrome, and transaminitis, which highlights the limited therapeutic window of FAP-directed immunotherapies [122,123]. Talabostat, a small-molecule FAP inhibitor with promising preclinical results, has been associated with adverse events in clinical trials, including edema and a reported Grade 5 event involving cytokine storm-related acute renal failure [124]. The TGF-β receptor inhibitor galunisertib has shown manageable toxicity and modest clinical benefit in pancreatic cancer, whereas significant hematologic toxicity has been reported in combination regimens including grade ≥3 neutropenia in 41.7% of patients in a Phase Ib trial of uterine or ovarian carcinosarcoma [125,126].

Collectively, these safety signals show that indiscriminate depletion of CAFs can disrupt important physiological processes of normal fibroblasts and other FAP-expressing cells, resulting in toxicities that may outweigh the therapeutic benefit. Future efforts should favor CAF subtype-specific targeting, transitory modulation over permanent ablation, and the use of predictive safety biomarkers to reduce toxicity concerns.

The main translational barriers should be addressed in the following order: first, confirm PNI-specific efficacy in nerve-containing models, because reduced tumor growth alone does not demonstrate inhibition of PNI; second, establish CAF selectivity and safety, given CAF heterogeneity and normal fibroblast functions; third, optimize delivery to the perineural niche and address compensatory signaling; and finally, develop biomarkers for patient selection and standardized PNI endpoints for clinical trials. This sequence prioritizes validation and safety before clinical translation.

## 5. Future Directions

### 5.1. Standardize PNI Assessment and Define CAF-Associated PNI Phenotypes

PNI has been demonstrated to be closely associated with poor prognosis and elevated local recurrence risk in a variety of cancers, including those of the head and neck, pancreas, and prostate. Nevertheless, extant research on PNI suffers from significant limitations that impede its precise clinical implementation. Firstly, the definition of PNI varies considerably across studies, and there is no universally accepted histopathological grading criterion applicable to different cancer types [21]. Secondly, radiological modalities such as MRI and CT are only capable of detecting macroscopic nerve involvement (such as nerve thickening or signal abnormalities). However, these modalities are insensitive to microscopic PNI [127]. Thirdly, the accuracy of histopathological examination depends on sample adequacy; inadequate sampling may miss focal perineural infiltration and lead to false negative results [128]. Moreover, PNI is not simply a process involving tumor cells and nerves but rather a dynamically evolving network that includes CAFs, SCs, immune cells (such as macrophages and T cells), and diverse signaling molecules. This intricate cellular and molecular complexity explains why PNI is clinically associated with high recurrence rates, severe pain, and poor therapeutic outcomes [129,130,131].

### 5.2. Development of More Physiologically Relevant PNI Animal Models

Current animal models of PNI face significant limitations in accurately recapitulating the complexity of human disease. Most existing models rely on direct injection of tumor cells into or adjacent to nerve structures, which does not fully mimic the natural multistep processes of PNI initiation, tumor–nerve crosstalk, and microenvironmental remodeling observed in patients. These models often fail to represent the dynamic interactions between tumor cells, nerves, stromal cells such as CAFs, and the immune microenvironment. Moreover, they inadequately capture the gradual and spontaneous nature of nerve invasion seen in clinical settings.

Future efforts should focus on establishing more physiologically relevant models that integrate the native TME, including CAFs and immune components, to better study the mechanisms of PNI and to evaluate targeted therapeutic strategies more accurately. In addition to improved animal models, such as orthotopic models, genetically engineered mouse models, and patient-derived xenograft models with preserved stromal components, complementary systems including 3D tumor–nerve-CAF co-culture models, organoid–nerve co-culture systems, and microfluidic platforms should also be developed. Ideally, these models should permit CAF depletion, CAF subtype-specific manipulation, lineage tracing, and real-time visualization of CAF-tumor–nerve interactions. Such models will be essential for determining whether CAFs actively drive PNI progression and for evaluating CAF-targeted therapeutic strategies in a clinically relevant context.

### 5.3. In-Depth Exploration of the Molecular Mechanism of CAFs in PNI

CAFs are well recognized as key regulators of tumor progression, and extensive research has elucidated their roles in promoting tumor proliferation, invasion, immune evasion, and therapy resistance. However, the specific functions and mechanisms of CAFs in PNI-positive tumors remain largely underexplored. Although the general interactions between tumor cells and nerve cells in PNI have been described, the contribution of CAF-secreted cytokines, chemokines, and growth factors to neural invasion lacks direct experimental evidence. Future research should focus on delineating the molecular and cellular mechanisms by which CAFs contribute to PNI, aiming to identify novel therapeutic targets for PNI-associated malignancies.

### 5.4. Understand the Dynamic Changes of CAF Subtypes in PNI

CAF subtypes and functional states are not static but highly plastic and can transition between phenotypes in response to dynamic cues from the TME, such as hypoxia, inflammation, and metabolic stress [132]. Current research lacks systematic exploration of CAF dynamics specifically in PNI-positive tumors. Most studies either focus on bulk CAF populations or characterize CAF subtypes in isolation, overlooking their transitional states and their context-dependent roles in neural invasion. Understanding how CAFs dynamically shift between subtypes and functional states during PNI is crucial, as it may reveal critical windows of vulnerability for therapeutic intervention. Interestingly, in vitro studies have shown that fibroblasts can be reprogrammed into Schwann-like cells under certain conditions [57,58,59]. Although this phenomenon has so far been observed only in non-cancer experimental systems, it raises the question of whether similar transitions might occur in CAFs within nerve-rich tumor environments. Future studies must move beyond static CAF classification and toward mapping their temporal and spatial evolution in PNI. Emerging approaches include single-cell omics, spatial transcriptomics, intravital imaging, and 3D nerve-tumor co-culture models to capture CAF plasticity and function in real time. However, substantial follow-up studies are still needed to validate CAF state transitions functionally and determine whether these dynamics are conserved across different PNI-prone cancer types.

### 5.5. Improve Clinical Interpretation of CAF Biomarkers Across Sample Types

The interpretation of CAF-associated biomarkers depends heavily on the biological sample being analyzed. Tissue-based analyses can define CAF populations in their spatial context. When combined with immunohistochemistry, single-cell RNA sequencing or spatial transcriptomics, they can distinguish CAF states according to marker expression and localization in relation to tumor cells, nerves and immune compartments. However, commonly used CAF markers such as FAP and α-SMA exhibit heterogeneity across CAF populations and are insufficient to define the entire CAF compartment. This complicates the interpretation of CAF phenotypes, even in tissue-based studies [133,134].

In contrast, blood-based analyses reflect CAF-associated signals in circulation rather than the spatial context of the TME. Circulating CAFs have been identified in peripheral blood using a combination of FAP and α-SMA expression. Detection was reported in 30 out of 34 patients with metastatic breast cancer, compared to three out of 13 patients with localized disease [135]. While this finding supports the feasibility of detecting circulating CAFs, their abundance does not directly reflect that of CAFs within the primary tumor or the perineural niche, nor their subtype composition or spatial organization.

This distinction is particularly relevant to PNI. In pancreatic cancer, CAF-derived extracellular vesicles carrying the PNI-associated transcript PIAT promote neural invasion through the PIAT–YBX1–m5C axis. Serum EV PIAT levels are also associated with the extent of neural invasion and patient prognosis. Therefore, serum EV PIAT levels offer a clinically accessible indicator of CAF-mediated PNI [30]. However, EVs in the serum represent a mixed pool of circulating vesicles, and studies of EV-based biomarkers are affected by vesicle heterogeneity, isolation methods, and difficulties in assigning vesicles to a specific cellular source. Consequently, it cannot be assumed that a circulating EV signal directly reflects the abundance or subtype distribution of CAFs surrounding invaded nerves.

Taken together, tissue- and blood-based CAF biomarkers provide complementary, albeit non-equivalent, information. Tissue profiling is better suited to defining the identity, subtype and spatial organization of CAFs within the perineural niche. In contrast, circulating CAFs or CAF-associated EV signals may be more useful as minimally invasive surrogate biomarkers for disease monitoring. Therefore, future studies should validate circulating CAF-associated markers against matched tumor tissues before using blood-based readouts to infer CAF states within the perineural microenvironment.

### 5.6. Targeted Therapeutic Development Against CAFs in PNI

Therapeutic strategies targeting CAFs have predominantly focused on inhibiting their roles in general tumor progression, including proliferation, invasion, and immune evasion. Several agents targeting CAF-derived signals, such as TGF-β, FAP, and CXCL12 pathways, have entered preclinical or clinical studies for various cancers [76,95,96,103,104,115]. However, the development of therapies specifically aimed at modulating CAF activity within the context of PNI remains extremely limited, and few drugs have been developed that specifically target the mechanisms of PNI [136]. Given the emerging evidence that CAFs actively contribute to PNI by remodeling the ECM, promoting tumor–nerve crosstalk, and shaping an immunosuppressive microenvironment, there is a pressing need to identify CAF-related molecular targets directly involved in PNI and to develop tailored therapeutic strategies. Future research should prioritize targets that are mechanistically linked to nerve invasion, such as CAF-derived neurotrophic signaling, CAF-mediated perineural ECM remodeling, chemokine-mediated tumor–nerve attraction, and CAF-driven immune suppression within the perineural niche. In addition, because CAFs are highly heterogeneous and may exert both tumor-promoting and tumor-restraining functions, non-selective CAF depletion should be approached with caution. More precise strategies, including reprogramming pro-PNI CAF states, blocking PNI-promoting CAF-derived signals, and combining CAF-targeted approaches with chemotherapy, radiotherapy, immunotherapy, or analgesic strategies, may provide more effective therapeutic opportunities. As discussed above, several CAF-related pathways may represent actionable candidates for PNI-directed therapy. The LIF/LIFR axis, CXCL12/CXCR4 signaling, and CAF-associated lactate metabolism are particularly relevant because they link stromal activity to SC dysfunction, neural invasion, immune exclusion, and metabolic reprogramming. These pathways also provide a rationale for combination strategies, such as pairing CXCL12/CXCR4 blockade with immune checkpoint inhibitors or integrating CAF-directed therapies with neuro-pain modulators. However, most of these approaches remain preclinical and require validation in PNI-specific models before clinical translation. Future research should therefore prioritize dissecting the unique features of CAFs in PNI-positive tumors to facilitate the development of more effective, nerve-invasion-specific anti-CAF therapies.

## 6. Conclusions

PNI represents a critical challenge in cancer management, and emerging evidence positions CAFs as important regulators of this process. Through subtype- and context-dependent stromal remodeling, CAFs orchestrate ECM reorganization, neurotrophic signaling, and SC activation, thereby facilitating tumor–nerve interactions, immune evasion, and metastatic progression. Current therapeutic approaches targeting CAFs, including inhibitors of surface markers, blockade of secreted factors, and modulation of intracellular signaling, have shown preclinical promise, yet clinical translation remains limited, and no CAF-targeted strategy has been established as a standard therapy specifically for PNI. Several major questions remain unresolved, including how CAF subtypes and functional states dynamically transition during PNI, how distinct CAF populations are spatially organized around invaded and non-invaded nerves, and which CAF-targeted strategies can achieve sufficient specificity, safety, and clinical efficacy in patients. Addressing these questions will require longitudinal and spatially resolved profiling of the perineural niche, physiologically relevant PNI models, mechanistic validation of CAF state transitions, and prospective clinical evaluation of CAF-directed biomarkers and therapeutic interventions. Such efforts may ultimately provide new opportunities to improve outcomes for patients with PNI-positive cancers.

## Figures and Tables

**Table 1:** Principal CAF subtypes and CAF-like states implicated in PNI: representative markers, reported functions, and PNI-specific evidence.

Principal CAF Subtype	Representative Markers	Reported Functions in PNI	PNI Evidence Level	References
**Myofibroblastic CAFs (myCAFs)/MMP2^+^ myCAF-like states**	Canonical myCAF markers: α-SMA/ACTA2, FAP, COL1A1. PNI-associated myCAF-like state: MMP2^+^ myCAF population in CRC; MMP-2^+^ perineural fibroblasts in OSCC.	myCAFs are mainly associated with TGF-β–driven myofibroblastic differentiation, fibrosis, collagen deposition, and ECM remodeling. In PNI, the strongest subtype-specific evidence supports MMP2^+^ myCAFs in CRC, whereas MMP2^+^ perineural fibroblasts in OSCC provide additional PNI-associated evidence. In CRC, MMP2^+^ myCAFs are enriched in PNI-positive tumors, with PRRX2 acting as a regulatory driver that promotes perineural infiltration through TGF-β/ECM-remodeling programs. In OSCC, MMP-2-expressing fibroblasts are detected around PNI-involved nerves, supporting an association with fibroblast-mediated matrix remodeling.	CRC—Direct PNI-specific evidence: Human CRC scRNA-seq and bulk transcriptomic evidence: 20 public CRC scRNA-seq datasets, TCGA PNI phenotype mapping, and BayesPrism deconvolution in TCGA plus three validation cohorts. OSCC—PNI-associated evidence: IHC staining of 12 OSCC patients showed MMP-2^+^ vimentin^+^ perineural fibroblasts at PNI-involved nerves.	[34,35,36,37]
**Inflammatory CAFs (iCAFs)/LIF-associated inflammatory CAF states**	Canonical iCAF-associated markers: Low α-SMA/ACTA2; high inflammatory cytokines and chemokines, including IL-6, CXCL1, CXCL8, and LIF. In OSCC, LIF-expressing CAFs were reported, but were not formally annotated as canonical iCAFs.	Canonical iCAFs shape inflammatory and immunosuppressive tumor microenvironments. In PDAC, SCs can induce an iCAF switch through IL-1α, suggesting that inflammatory CAF-like states may participate in neuro-stromal remodeling. In OSCC, CAF-derived LIF promotes PNI by inducing SC inactivation, reducing SC proliferation and migration, disrupting myelin homeostasis, and aggravating nerve injury.	PDAC—PNI-associated evidence: Human scRNA-seq and spatial transcriptomic analyses showed enrichment of iCAF signatures in nerve-proximal, SC-rich regions; in vitro and in vivo studies further demonstrated that SC-derived IL-1α can induce an iCAF-like shift in human PDAC-derived CAFs.	[31,34,38,39]
**Antigen-presenting CAFs (apCAFs)**	MHC class II-related molecules, including HLA-DRA/H2-Aa and CD74; SAA3 has also been reported in mouse pancreatic apCAF populations.	apCAFs primarily regulate adaptive immune responses through antigen-presentation-related programs, particularly by interacting with CD4^+^ T cells. No direct PNI-specific function has been established.	Indirect/extrapolated evidence. Supported by pancreatic cancer TME studies of antigen presentation and T-cell regulation; no direct PNI-specific functional evidence has been established.	[35,40,41]
**Metabolically active CAF states (meCAFs)**	Metabolism-associated markers: LDHA, GLUT1, and other glycolysis-related genes. PNI-associated metabolic CAF-like features: glycolytic/high-lactate CAF program in PDAC.	meCAFs support tumor progression by producing metabolic intermediates and shaping tumor metabolism and immune modulation. Direct evidence for canonical meCAFs in PNI remains limited. However, in PDAC, PNI-associated CAFs display enhanced glycolysis and generate a high-lactate microenvironment; CAF-derived lactate induces H3K18 lactylation in tumor cells and activates neural invasion-related genes such as L1CAM and SLIT1, thereby promoting PNI.	Direct PNI-specific functional evidence in PDAC: Human PDAC samples showed glycolytic/high-lactate PNI-associated CAFs, with in vitro and mouse-model validation demonstrating that CAF-derived lactate promotes PNI through H3K18 lactylation and L1CAM/SLIT1 activation. This evidence supports a PDAC-specific metabolic CAF state rather than a universally conserved canonical meCAF subtype.	[29,42,43,44]

**Table 2:** Therapeutic agents targeting CAFs and their potential relevance to PNI.

Target	Drug(s)	Indication(s)	Development Stage	Evidence Relevant to PNI	References
**FAP**	Talabostat	Metastatic colorectal cancer; preclinical inhibition of CAF-driven ECM remodeling	Phase II (limited efficacy)	Indirect: no direct PNI model evaluation.	[95]
	Anti-FAP-PE38 immunotoxin	Breast cancer models; reduces tumor growth and enhances immune infiltration	Preclinical	Indirect: breast tumor and immune effects; no direct PNI model evaluation.	[96]
**PDGFR-β**	TKIs (imatinib, sorafenib, etc.)	GIST, RCC, leukemia; used in broad solid tumor therapy	FDA-approved for specified cancer indications	Indirect: stromal signaling rationale; no PNI-specific efficacy data.	[97]
**PDGFR-α**	Olaratumab	Soft tissue sarcoma; withdrawn after Phase III failure	Withdrawn post–Phase III	Indirect: sarcoma studies; no PNI-specific efficacy data.	[98,99]
**CCR2**	PF-04136309	Pancreatic cancer with FOLFIRINOX; improved tumor control	Phase I	Indirect: pancreatic cancer trial without PNI-specific efficacy endpoints.	[100]
**CCL2**	Carlumab	Castration-resistant prostate cancer	Phase I/II	Indirect: pathway relevance to PNI; antibody not tested directly in PNI.	[101]
**IL-6**	Tocilizumab	CRC xenografts	Preclinical (CRC efficacy)	Indirect: CRC xenograft evidence; no direct PNI evaluation.	[102]
**TGF-β**	Galunisertib	Neuroblastoma models	Preclinical	Indirect: antitumor evidence; no direct PNI validation.	[103]
	SB-431542	Pancreatic cancer cell models	Preclinical	Indirect: tumor signaling studies; no direct PNI validation.	[104]
**CAF-derived EVs/PIAT**	Targeting YBX1, m5C modification, or EV secretion inhibitors	Studied in pancreatic cancer; CAF EVs deliver PIAT to promote YBX1 stabilization	Preclinical	PNI-related mechanism: PIAT/YBX1 studied in neural-invasion models; efficacy of generic EV inhibitors is extrapolated.	[30]
**LIF**	EC330	Oral squamous carcinoma, breast, pancreatic, endometrial cancer	Preclinical	PNI-related cellular evidence: EC330 restored SC function; direct drug-mediated reduction in PNI in vivo remains unproven.	[31,105]
**LOXL2**	Simtuzumab	Fibrosis, advanced solid tumors, metastatic CRC and pancreatic adenocarcinoma	Phase I/II (failed to show clinical benefit)	Indirect: ECM/stromal rationale; no direct PNI evaluation.	[106,107,108]
**YAP1/TEAD1**	Experimental YAP1 inhibitor; verteporfin	Prostate cancer PNI model; glioblastoma models	Preclinical oncology studies; verteporfin approved for ocular use	Direct: experimental YAP1 inhibition reduced invasion toward DRG. Indirect: verteporfin in glioblastoma models.	[109,110]
**JAK2/STAT3**	AG490	Gastric cancer	Preclinical	Indirect: inhibition of CAF-induced gastric cancer cell migration and invasion; no direct PNI evaluation.	[111]
	STAT3 siRNA	Non-small cell lung cancer (NSCLC) and breast cancer models	Preclinical	Indirect: invasion assays and PNI association; no direct drug evaluation in a PNI model.	[112,113]
**PI3K/AKT**	MK-2206	NSCLC models	Preclinical	Indirect: no anti-PNI evidence; increased invasion/metastasis in selected lung cancer models.	[114]

## Data Availability

No new data were created or analyzed in this study. Data sharing is not applicable to this article.

## Abbreviations

PNI	Perineural invasion
CAFs	Cancer-associated fibroblasts
HNSCC	Head and neck squamous cell carcinoma
CRC	Colorectal cancer
OS	Overall survival
DSS	Disease-specific survival
DFS	Disease-free survival
TME	Tumor microenvironment
TGF-β	Transforming growth factor beta
TGF-β1	Transforming growth factor beta 1
SCs	Schwann cells
ECM	Extracellular matrix
MMPs	Matrix metalloproteinases
NGF	Nerve growth factor
EMT	Epithelial–mesenchymal transition
PIAT	Perineural invasion-associated transcript
OSCC	Oral squamous cell carcinoma
LIF	Leukemia inhibitory factor
LIFR	Leukemia inhibitory factor receptor
myCAFs	Myofibroblastic CAFs
iCAFs	Inflammatory CAFs
apCAFs	Antigen-presenting CAFs
meCAFs	Metabolic CAFs
FAP	Fibroblast activation protein
Saa3	Serum amyloid A3
LDHA	Lactate dehydrogenase A
GLUT1	Glucose transporter 1
PDAC	Pancreatic ductal adenocarcinoma
FGF	Fibroblast growth factor
DRG	Dorsal root ganglion
NE	Norepinephrine
EVs	Extracellular vesicles
LOX	Lysyl oxidase
CGRP	Calcitonin gene-related peptide
PDGFR-β	Platelet-derived growth factor receptor-beta
α-SMA	Alpha-smooth muscle actin
ACTA2	Actin alpha 2, smooth muscle
COL1A1	Collagen type I alpha 1 chain
IL	Interleukin
CXCL	C-X-C motif chemokine ligand
CXCR	C-X-C motif chemokine receptor
CCL2	C-C motif chemokine ligand 2
CCR2	C-C motif chemokine receptor 2
Tregs	Regulatory T cells
MHC	Major histocompatibility complex
YBX1	Y-box binding protein 1
m5C	5-Methylcytosine
LOXL2	Lysyl oxidase-like 2
YAP1	Yes-associated protein 1
TEAD1	TEA domain transcription factor 1
JAK2	Janus kinase 2
STAT3	Signal transducer and activator of transcription 3
PI3K	Phosphoinositide 3-kinase
AKT	Protein kinase B
NSCLC	Non-small cell lung cancer
CAR-T	Chimeric antigen receptor T cell
TKI	Tyrosine kinase inhibitor
TrkA	Tropomyosin receptor kinase A
SPHK1	Sphingosine kinase 1
S1P	Sphingosine-1-phosphate
L1CAM	L1 cell adhesion molecule
SLIT1/2	Slit guidance ligand 1/2
scRNA-seq	Single-cell RNA sequencing
TCGA	The Cancer Genome Atlas
IHC	Immunohistochemistry
Th1/Th2	Type 1/type 2 helper T cells
